# The Role of the Innate Immune System in Alzheimer's Disease and Frontotemporal Lobar Degeneration: An Eye on Microglia

**DOI:** 10.1155/2013/939786

**Published:** 2013-07-18

**Authors:** Elisa Ridolfi, Cinzia Barone, Elio Scarpini, Daniela Galimberti

**Affiliations:** Neurology Unit, Department of Pathophysiology and Transplantation, University of Milan, Fondazione Cà Granda, IRCCS Ospedale Maggiore Policlinico, Via F. Sforza 35, 20122 Milan, Italy

## Abstract

In the last few years, genetic and biomolecular mechanisms at the basis of Alzheimer's disease (AD) and frontotemporal lobar degeneration (FTLD) have been unraveled. A key role is played by microglia, which represent the immune effector cells in the central nervous system (CNS). They are extremely sensitive to the environmental changes in the brain and are activated in response to several pathologic events within the CNS, including altered neuronal function, infection, injury, and inflammation. While short-term microglial activity has generally a neuroprotective role, chronic activation has been implicated in the pathogenesis of neurodegenerative disorders, including AD and FTLD. In this framework, the purpose of this review is to give an overview of clinical features, genetics, and novel discoveries on biomolecular pathogenic mechanisms at the basis of these two neurodegenerative diseases and to outline current evidence regarding the role played by activated microglia in their pathogenesis.

## 1. Introduction

Dementia is a chronic or progressive loss of cortical and subcortical functions resulting in cognitive decline, accompanied by disturbances of mood, behavior and personality, and synaptic loss [1]. Alzheimer's disease (AD) is the most prevalent dementia in the elderly, whereas the most common type of dementia in the presenile population (<65 years) is frontotemporal lobar degeneration (FTLD).

Alzheimer' s disease affects up to 75% of the more than 35 million people suffering from dementia worldwide, and the prevalence is believed to double every 20 years [2]. FTLD instead represents nearly 20% of cases of early-onset dementia [3].

Both AD and FTLD are characterized by insoluble filamentous aggregates in the brain. They share this feature with Parkinson's disease, Lewy body dementia, and Creutzfeldt-Jakob disease. In particular, AD pathology is characterized by amyloid beta (A*β*) plaques and tau-containing neurofibrillary tangles (NFTs). The abnormal protein accumulation triggers a brain inflammatory reaction, inducing the production of a series of proinflammatory mediators and microglial activation [4]. Chronic microglial activation may contribute to the development and progression of neurodegenerative disorders, such as AD and FTLD.

The purpose of this review is to describe the main clinical and biomolecular features of AD and FTLD and to characterize the role of neuroinflammation in the pathogenesis of these diseases, with particular interest on the role played by microglia, which represents the immune system of the brain. 

## 2. Clinical Features of Alzheimer's Disease and Frontotemporal Lobar Degeneration 

### 2.1. Alzheimer's Disease

Alzheimer's disease is a progressive neurodegenerative disease, which is characterized by cognitive decline due to neuronal loss [5–7]. The hallmarks of AD are the extracellular A*β* plaque deposition (named senile plaques) and the intraneuronal NFTs of hyperphosphorylated tau protein [8]. Deposition of A*β* seems to be the first biological process during the pathogenesis of AD, beginning many years before the appearance of symptoms. Also tau deposition in the brain, despite occurring later than A*β*, starts before clinical onset of the disease. These changes are well reflected in the cerebrospinal fluid (CSF), in which increased levels of total tau and phosphorylated tau as well as decreased A*β* levels are altered early during the pathogenesis [9]. Several studies showed in fact that changes in these biomarkers can be seen in the preclinical stage of the disease (mild cognitive impairment, (MCI)), and this can be useful to establish MCI subjects that likely will turn into AD [10]. In a minority of cases, very often with an early onset (from the forth decade), AD is transmitted with an autosomal dominant pattern of inheritance. These cases are caused by autosomal dominant mutations in specific genes, including amyloid precursor protein (*APP*) and presenilin (*PSEN*) 1 and 2 [11]. However, except for *PSEN2*, all the mutations are fully penetrant. The majority of cases have a multifactorial pathogenesis, resulting from the combination of several genetic and environmental factors. The main gene associated with sporadic AD is the apolipoprotein E gene (*APOE*) [12].

### 2.2. Frontotemporal Lobar Degeneration

Frontotemporal lobar degeneration comprises a spectrum of clinical syndromes and is pathologically and genetically heterogeneous. The disease onset occurs between 45 and 65 years, and the prevalence is equal between men and women (see [17] for review). The most frequent clinical features are progressive changes in behavior, executive dysfunction, and language impairment.

Frontotemporal lobar degeneration can manifest with two major syndromes: behavioral variant frontotemporal dementia (bvFTD) and primary progressive aphasia (PPA). bvFTD is characterized by changes in behavior and personality, such as disinhibition, apathy, and loss of empathy, leading to a loss of social competence. bvFTD has been associated with symmetrical ventromedial frontal, orbital frontal, and insular atrophy and left anterior cingulate atrophy [13]. “The International Behavioral Variant FTD Criteria Consortium” established international consensus criteria for bvFTD: a diagnosis of bvFTD is based upon a three-tier, hierarchical classification system into “possible,” defined by clinical criteria, “probable,” supported by neuroimaging data, and “definite” bvFTD, confirmed by neuropathological evidence or the identification of a pathogenic mutation [14]. The clinical feature of PPA [15] is pronounced impairment in language, which may consist in deficits in language articulation, object-naming, syntax, and word comprehension. Primary progressive aphasia is further categorized into progressive nonfluent aphasia (PNFA), characterized by expressive or motor speech deficits with predominantly left perisylvian atrophy and semantic dementia (SD), described by a loss of semantic knowledge with associated atrophy of the left greater than the right anterior temporal lobes [16]. A third newly described subtype is named logopenic aphasia [15].

Furthermore, there is a significant clinical, pathological and genetic overlap between FTLD and amyotrophic lateral sclerosis (ALS). FTLD-ALS patients have a poor diagnosis with a mean survival of 2-3 years from the onset of first symptoms [16, 17]. Other diseases are closely related to FTLD, such as progressive supranuclear palsy, corticobasal syndrome, and FTD with parkinsonism [13].

The neuropathology of FTLD can be divided into four subtypes, according to its histology: with tau deposits (FTLD-tau); with tau-negative, but with ubiquitin (FTLD-ubiquitin or FTLD-U) and TAR DNA binding protein (TDP)-43 positive inclusions (FTLD-TDP); with neuronal intermediate filament inclusions and cases with no detectable inclusions [18]. There are also a considerable number of TDP-43 negative FTLD-U cases with inclusions of fused-in-sarcoma protein (FUS), referred to as FTLD-FUS [13].

## 3. Genetics

### 3.1. Autosomal Dominantly Inherited Alzheimer's Disease

The *APP* gene is localized in the chromosome 21 and encodes for A*β* precursor, a transmembrane polypeptide of 770 amino acids. The release of A*β* follows at least two APP cleavages, processed by different classes of secretases. The first cleavage occurs within the extracellular domain by *α*- or *β*-secretase, and the second proteolytic cut takes place in the transmembrane region by a third secretase, known as *γ*-secretase. In the amyloidogenic pathway, *APP* is processed sequentially by *β*- and *γ*-secretase, with the generation of A*β*40-42 fragments, which aggregate and form senile plaques [19–21]. The most common *APP* mutations occur in the transmembrane domain or in the *γ*-secretase cleavage site, leading to an increased A*β* production [20, 21]. Similarly, in the Down syndrome, the presence of a third *APP* copy gene causes an A*β* overproduction, which explains why the patient with Downs syndrome develops AD pathology in their brains [22]. Finally, substitutions that take place within the A*β* peptide result in a peptide that is more prone to cluster together and to form aggregates [20, 21]. The other two genes involved in familial AD are components of *γ*-secretase complex, known as *PSEN1*, and its homologous *PSEN2*. *PSEN1*, which is in the chromosome 14, consists of 12 exons encoding for a transmembrane protein [23]. Its homologous *PSEN2* is located in the chromosome 1, and encodes also for a transmembrane protein consisting of 12 exons [24]. PSEN1, or its isoform PSEN2, forms the catalytic core of *γ*-secretase complex together with nicastrin, anterior pharynx-defective 1, and presenilin enhancer 2. Most of *PSEN1 *mutations are missense mutations, which lead to an altered cleavage site in the *APP* sequence. The patients carrying these mutations have an autosomal dominant inheritance form of AD with a full penetrance and an age of onset of about 30 years old [11, 25]. Conversely, *PSEN2* mutation is an uncommon cause of AD with an incomplete penetrance and later onset [11]. Mutations in *PSEN1/2* genes cause an increased A*β*40/A*β*42 ratio with increased A*β*42, that is, more incline to aggregate than A*β*40 [20, 21]. Patients with different mutations in the same gene show heterogeneous clinical features [26].

### 3.2. Autosomal Dominantly Inherited Frontotemporal Lobar Degeneration

 Frontotemporal lobar degeneration has a strong genetic component, demonstrated by the fact that about 40% of FTLD patients have a positive family history of dementia [27]. Several genes have been recognized to play a role in autosomal dominant FTLD: microtubule-associated protein tau (*MAPT*), progranulin (*GRN*), and *C9ORF72*. In a minority of FTLD cases, valosin-containing-protein-(*VCP-*) 1, transactive DNA-binding protein (*TDP-43*), *FUS*, and chromatin-modifying protein 2B (*CHMP2B*) genes have been found to be responsible for the disease [3]. 

The first mutations causing FTD with parkinsonism (FTDP-17) were first found in chromosome 17 *MAPT* [28], which encodes for the tau protein, critical for microtubule assembly and stabilization in neurons. At present, more than 40 mutations have been identified in 134 pedigrees (http://www.molgen.vib-ua.be/ADMutations/default.cfm?MT=1&ML=0&Page=ADMDB). Mutations are predominantly clustered in exons 9–13 of the microtubule-binding region near the alternatively spliced exon 10 [29] and mostly modify the normal function of tau [30, 31].

Mutations in a second gene in chromosome 17, named progranulin (*GRN*), were discovered in 2006 [32, 33].* GRN* mutations cause about 5–10% of all FTLD cases, varying depending on the population considered (see [17] for review). To date, 69 different *GRN* mutations have been described in 231 families (http://www.molgen.vib-ua.be/ADMutations/default.cfm?MT=1&ML=0&Page=ADMDB). *GRN* mutations, which include frameshift, splice-site, and nonsense mutations, are distributed across the complete coding region and splice sites of the gene. These are loss-of-function mutations, which lead to reduced functional protein, resulting in haploinsufficiency [13]. 

Recently, a hexanucleotide repeat expansion in the noncoding region of *C9ORF72* has been recognized as the genetic abnormality on chromosome 9p21 underlying the majority cases of familial FTLD [3]. Majounie and colleagues observed pathological *C9ORF72* repeat expansions in 11.4% of 1,381 FTLD patients of European origin, rising to 24.8% in familial patients, whereas a *C9ORF72* repeats expansion frequency of 6.0% in sporadic FTLD [13]. The chromosome 9 expansion seems to mediate neurodegeneration through an RNA-mediated mechanism. Wild-type alleles contain less than 30 repeats, while mutated alleles have hundreds to thousands repeats [34]. 

The frequency of the hexanucleotide repeat expansions has been recently determined in a large population of 651 FTLD patients and the clinical characteristics of carriers and noncarriers compared. 39 FTLD patients (6%) presented the pathogenic repeat expansion: 24 of these patients had a positive family history for dementia and/or ALS (61.5%). The presentation with late-onset psychosis was more frequent in carriers than noncarriers, as well as the presence of cognitive impairment at onset. These data confirmed that the repeat expansion in *C9ORF72* is a common cause of FTLD and, importantly, it is often associated with late-onset psychosis and memory impairment [35].

### 3.3. Sporadic Alzheimer's Disease: Genetic Risk Factors

A number of genetic variants contribute to the risk of developing sporadic AD. The gene that is strongly associated with AD is *APOE*. This gene is located in chromosome 19 and consists of 4 exons, which encodes for a protein of 229 amino acids. There are three APOE isoforms: *ε*2, *ε*3, and *ε*4. The *ε*3 isoform is the most common allele among populations, while the *ε*2 allele is found in only 1–5% of people and likely plays a protective role, by decreasing AD risk [36]. The *ε*4 allele is found in the 50% patients affected by AD and confers a threefold increased risk for AD development [37]. A*β* trafficking, metabolism, and accumulation are regulated by APOE in a different manner by the three APOE isoforms. Perhaps this is the reason why the *ε*4 allele confers a high AD risk, while the *ε*2 allele is a protective factor [38–40]. Even though APOE *ε*4 itself cannot be the cause of AD [41], the *ε*4 isoform leads to an earlier onset of symptoms in *APP* or *PSEN1* mutation carriers [42]. Many additional risk variants have been described on a candidate-based hypothesis. In the last few years, Genome Wide Association Studies (GWAS), carried out in large populations, identified novel risk genes, including complement receptor type I (*CRI*), phosphatidyl inositol binding clathrin assembly protein (*PICALM*), clusterin (*CLU*), and bridging integrator 1 (*BIN1*).

### 3.4. Susceptibility Genes and Risk Loci in Frontotemporal Lobar Degeneration

Little is known about susceptibility genes contributing to the risk of developing FTLD. 

There has been so far only one GWAS in FTLD-TDP, which identified a possible susceptibility locus, which encompasses the gene *TMEM106B* on chromosome 7p21 [43]. The study identified three significantly associated single nucleotide polymorphisms (SNPs), which seemed to be related to an increased expression of *TMEM106B*, a condition that could be possibly involved in the pathogenesis of FTLD-TDP [43]. 

Finch et al. tried to replicate the association of *TMEM106B* SNPs, using a large series of patients with FTLD with and without *GRN* mutations. They also performed *in vivo* studies in plasma and peripheral blood to test the hypothesis that *TMEM106B* SNPs regulate GRN expression levels and influence FTLD risk by modulating GRN expression. The authors found that *TMEM106B* SNPs significantly reduced the disease penetrance in patients with *GRN* mutations, potentially by modulating GRN levels [44]. 

Apart from this GWAS study, Rademakers and colleagues demonstrated that a common genetic variant (rs5848), located in the 3′-untranslated region of *GRN* in a binding-site for microRNA-659, was a major susceptibility factor for FTLD-U [34]. Another variant, in the first intron of *GRN*, was also reported to be associated with FTLD in another cohort of patients [45]. Details on additional candidate-based genes associated are reviewed in [46]. 

## 4. Microglia and Neuroinflammation

 Microglia are the unique resident macrophages of the CNS, representing about 5–10% of the adult brain cell population [47]. In healthy conditions, microglia have a ramified morphology with a small, round soma and numerous branching processes [48]. During development, microglial cells are involved in different processes, such as clearance of dying or dead cells, elimination of excess axons, promotion of neuroaxonal growth, axonal guidance, neuronal differentiation, regulation of embryonic cortical precursor cell development, astrocyte proliferation, and angiogenesis. They are also able to release a variety of cell signaling factors like neurotrophins and extracellular matrix components [47]. 

A number of pathologic events, including altered neuronal function, infection, injury, and inflammation, rapidly activate microglia. Activated microglia change from a ramified to a hyperramified phenotype, proliferate, migrate to the site of damage, and secrete both cytotoxic and neurotrophic factors [49]. As the main cells of innate immunity of the CNS, microglia constitutively express the most important receptors (MHC I and II, chemokine receptors) at low levels [50]. During activation, the immunologically relevant molecules are upregulated and the appropriate antigen presented via MHC II [51]. Moreover, microglial cells can cross present exogenous antigens on MHC I to CD8^+^ T cells [52]. 

Microglia can present two distinct molecular phenotypes and effector functions depending on the activation pathway. The M1 phenotype is induced by classic activation of microglia and is characterized by production of proinflammatory cytokines, such as IL-1*β* and TNF-*α*, and free radicals such as reactive oxygen species (ROS). It plays a central role in the defense against pathogens and tumor cells but can also damage healthy cells, like neurons and glial cells. The alternative M2 anti-inflammatory phenotype is induced by IL-4 and IL-13 and expresses CD206 and arginase 1, which downregulate inflammation and promote tissue remodeling/repair and angiogenesis [47]. 

While short-term microglial activity has generally a neuroprotective role, chronic activation has been implicated in the pathogenesis of neurodegenerative disorders. The exact mechanism leading to microglial overactivation is still not fully understood, but glial-neuronal crosstalk seems to be central, as well as microglia and astrocyte interaction. Proinflammatory cytokines secreted by activated microglia inhibit astrocyte gap junction communication, which influences the role of astrocytes in providing neuronal support [50]. Moreover, activated microglia can release the neurotransmitter glutamate, which trigger excitotoxic neurodegeneration and cell death of astrocytes and oligodendrocytes [47]. 

### 4.1. Microglia Activation and the Role of Inflammation in AD

Lines of evidence of inflammatory involvement in AD were first observed by Alois Alzheimer in autopsied brains from patients with AD [53, 54]. This hypothesis is supported by the epidemiologic studies which show that patients treated with nonsteroidal anti-inflammatory drugs (NSAIDs) had a reduced incidence of AD [55–57]. Later, neuron models demonstrated that the metabolites and products of inflammatory reaction, were neurotoxic [58, 59]. These products of inflammatory reactions may initiate and promote neuronal degeneration in AD [60].

Neuroinflammation depends essentially on microglia and astrocytes activation that respond to various injuries and stimulations, through the expression of inflammatory factors such as cytokines and chemokines, and the release of reactive oxygen species (ROS) and nitric oxide (NO) that finally cause oxidative stress [61–64]. Many studies, in fact, show that oxidative stress responsive transcription factors, such as NF-*κ*B and CHOP, are directly linked with the inflammatory pathway by regulating proinflammatory genes [65–67].

 Cytotoxic activation and inflammatory factors stimulate glial cells to the release of several proinflammatory signals generating a vicious circle between neuroinflammation and oxidative stress that results in a self-sustaining inflammatory condition [68].

This inflammatory state could, moreover, modify intercellular communication through the deregulation of gap junction channel and hemichannel [69]. In AD, astrocytes show a decreased gap junctional intercellular communication (GJIC) and an increased hemichannel functionality [70–74]. During AD progression, A*β* accumulation increases hemichannel activity, which generates Ca^2+^ diffusion also through GJIC. This influx raises the intracellular Ca^2+^ concentration in the neighbors astrocytes cells allowing the release of glutamate and activation of neuronal NMDA receptors [75]. The hemichannel activation, instead, drives the release of ATP that finally activates the purinergic receptor (P2). P2 and NMDA affect electrochemical and Ca^2+^ imbalance in neurons, which leads finally to cell death [76]. Similarly, decreased GJIC may defend the physiological intercellular diffusion of nutrient and metabolites, essential for growth and survival of neurons, generating cell death. On the other hand, the decreased GJIC may be a defensive mechanism to avoid the diffusion of death signal and toxic molecules through cells [77]. In this contest, even though the main AD hallmarks are senile plaques and neurofibrillary tangles, several recent studies have showed that activated microglia are one of the principal players of neuroinflammation, together with A*β* plaques deposition or astrocytes stimulation [78].

Wisniewski et al. [79], by electron microscopic analysis, observed that at least 80% of A*β* plaques colocalize with activated microglia in the brain of AD patients [79, 80]. *In vivo* and *in vitro* experiments suggest that microglia, recruited in A*β* plaques site, are able to surround and phagocytize A*β* peptides. This mechanism is regulated by Toll-like receptor, suggesting an impairment of the immune system in AD pathogenesis. In the same way, astrocytes cells, which provide trophic and metabolic support to neurons, also regulate phagocytic activity of microglia cells [81–83]. The lines of evidences show that under chronic inflammatory condition, astrocytes are unable to preserve the scavenger role of microglia suggesting an impairment of this physiological mechanism in AD patients [84]. 

A*β* plays also an important role in the regulation of proteasome activity witch is essential for the degradation of ubiquitin-conjugated proteins [85]. The deregulation of this mechanism seems to be involved in the neuroinflammation neurodegeneration process, and A*β* is one of the factors that could inhibit proteasome functions [86–90]. Moreover, A*β* deposition is responsible for microglia activation in a way strictly dependent on amyloid load; evidence of this comes from many experiments, where *APP* gene disruption reduces microglia activation and decreases neuroinflammation [91].

A*β* contributes to enhance inflammatory response by NF-*κ*B stimulation, a nuclear factor implicated in cytokine production and also regulates the extracellular signal-regulated kinase (ERK) and mitogen-activated protein kinase (MAPK) pathway that leads to cytokines and chemokine production [92, 93]. Several proinflammatory cytokines and chemokines have been found dysregulated in AD patients [94]. IL-1, IL-6, and TNF-*α*, which are proinflammatory cytokines, were found upregulated in AD patients [95]. In particular, IL-1 induces microglia and astrocytes activation, enhancing inflammation; moreover, it stimulates acetylcholinesterase and iNOS enzymatic activity [96–98]. IL-6 supports astrogliosis and stimulates the release of acute phase protein [99–101]. In addition, TNF-*α* expression is regulated by CD40L. Activated microglia, treated with CD40L, lead to an increased TNF-*α* level that results in neuron injury. The evidence comes from the APP mice deficient for CD40 that shows decreased microglia activation and reduction of inflammatory response [102]. *In vitro* analysis showed that A*β* stimulates the secretion of specific chemokines, such as CCL2 and CCL3. In AD patients, where their levels are elevated, CCL2 and CCL3 recruit astrocytes and microglia cells to A*β* plaques site increasing neuroinflammation [103, 104]. Cytokines and chemokines are altered in CSF from patients compared with controls. Galimberti et al. showed that IL-11 levels are increased in CSF of mild AD and FTLD patients and positively correlated with the Mini Mental State Examination (MMSE) score. On the contrary, MCP1 and IL-8 levels were increased in all AD and in almost FTLD patients. Moreover, IP10 concentration was elevated in CSF from AD patients as well as subjects with MCI but was unchanged in FTLD patients [105, 106]. By putting these data together, these studies suggest that IP-10 probably is involved only in AD pathology. On the contrary, MCP1 and IL-11, which were found upregulated in FTLD and others neurodegenerative disease, may be implicated in a common step shared by these pathologies. The positive correlation between high MMSE score and the IL-11/IP-10 peaks detected in CFS suggests that up-regulation of these cytokines is an early event in the pathogenesis of AD/FTLD, that is, not observed in the late stages of the disease, implying that the high level of proinflammatory cytokine represents an early and transitory effort of immune cells to restore brain health [105, 107]. Additionally, the complement system is active in the early stage of the disease. In fact, A*β* is able to activate the complement system by alternative pathway [108]. Nonetheless, the complement activation produces inflammation and cell damage; studies in mice models showed that the complement system had also neuroprotective roles. These studies demonstrated that the complement complex inhibition increased A*β* deposition, suggesting that the complement complex is essential for clearance of apoptotic cells, debris cells, and toxic protein aggregates [109–111].

Recent investigations showed that activated microglia and inflammation are not always detrimental but can have a reparative role in neuronal damage. For example, CD45, a bound protein-tyrosine phosphatase, is highly expressed in activated microglia of AD patients rather than controls [112]. Tan et al. inhibited CD45 activity to investigate its role in reactivity of microglia to the A*β* protein [113]. The results showed an increased level of TNF-*α* and other toxic molecules, which generated neuron damage. The use of CD45 agonist decreased TNF-*α* expression and oxide nitric production, downregulating microglial activation. The conclusion is that CD45 could play beneficial and protective effects in AD [114]. 

Other studies show that sometimes proinflammatory cytokines promote beneficial neuroinflammation, decreasing AD mice amyloidosis. Shaftel et al. [115] created a transgenic mouse IL-*β* ^XAT^ to study the role of IL-1*β* in neuroinflammation. This transgenic mouse overexpressed IL-*β* in a specific temporal and spatial manner following FIV-Cre injection. IL-*β* ^XAT^ mouse showed a high neuroinflammation caused by astrocytes and microglia activation. APP mice were crossed with IL-*β* ^XAT^ mice, showing a drastic reduction of A*β* plaques and an increased phagocytosis activity [115]. These data suggest that IL-*β* expression leads to a beneficial neuroinflammation that enhances A*β* removal. Other investigators obtained the same results by expressing IFN-*γ*, in TgCRND8 mice, a mouse model of cerebral amyloidosis. The analysis, after IFN-*γ* injection, showed a remarkable decrease of A*β* plaques [116]. Taken together, these data suggest that certain forms of inflammation and microglia activation are quite helpful in neurodegenerative pathology, such as AD [117].

### 4.2. Microglia, Tau, and Progranulin: Role in FTLD

Cagnin et al. [118] first observed microglial activation in FTLD. They used positron emission tomography and a marker of “peripheral benzodiazepine sites,” [^11^C] (R)-PK11195, which is upregulated on activated microglia during progressive tissue pathology [119]. They demonstrated an increased binding of [^11^C] (R)-PK11195 in the typically affected frontotemporal brain regions, which implied the presence of activated microglia response reflecting progressive neuronal degeneration [118]. 

Interestingly, activated microglia express progranulin [120–122]. Progranulin expression greatly increased in response to experimental traumatic spinal cord injury in a mouse model, and microglia were the primary sources of progranulin after injury [120]. Tanaka et al. [121] demonstrated that progranulin deficiency induced exacerbated inflammatory responses associated with activated microglia, such as excessive increase of CD68-positive cells, which is a marker for activated microglia, and TGF*β*-Smad3 signaling, a central mediator initiating formation of the glial scar [123], excessive protein oxidation and laminin immunoreactivity after traumatic brain injury [121].

Yin et al. [124] tried to understand the mechanism by which loss of function mutations in *GRN* cause FTLD. They generated conditional *GRN* knock-out mice, with the expectation that *GRN *gene deletion might be embryonic lethal. Instead, it was not the case. Initially, they observed that *GRN*-deficient macrophages produced more proinflammatory cytokines and chemokines, such as CCL2, CXCL1, IL-6, IL-12p40, and TNF, but less anti-inflammatory cytokine IL-10 compared to wild-type (wt) macrophages, when exposed to bacterial lipopolysaccharide. However, *GRN*-deficient mice failed to clear bacterial infection as fast as wt mice and were characterized by an exaggerated inflammatory tissue damage. Immunostaining of brain sections for CD68 revealed greater activation of microglia with age in *GRN*-deficient mice than wt mice. Moreover, *GRN*-deficient microglia responded to inflammatory stimuli by becoming more cytotoxic than wt microglia, and *GRN*-deficient neurons were more susceptible than wt to damage by activated microglia and by certain cytotoxic stresses, such as depletion of glucose and oxygen. They also showed enhanced hippocampal ubiquitin immunostaining and increased phosphorylation of TDP-43 in the hippocampus and thalamus of old *GRN*-deficient mice. The authors thus hypothesized that FTLD may arise from the congruence of two independent phenotypes of *GRN* insufficiency: deregulated inflammation and increased neuronal vulnerability to damage [124]. In another study, the same authors assessed the behavioral profile of *GRN*-deficient mice from 1 to 18 months (mo) of age, finding impairment in social recognition tasks. Behavioral deficits appeared early in the disease (at 1 mo of age), and they were still present at 18 mo of age. These findings were consistent with the behavioral and personality changes observed in FTLD patients. A possible mechanism underlying *GRN*-deficiency-linked behavioral deficits is that the anti-inflammatory action of *GRN* may be indispensable for a balanced immune response in the brain [125].

Martens et al. [126] demonstrated that the loss of *GRN* resulted in increased neuron loss in response to injury in the CNS. When exposed acutely to 1′-methil-4-(2′-methylphenyl)-1,2,3,6-tetrahydrophine (MPTP), a neurotoxin that targets the dopaminergic neurons of the substantia nigra pars compacta (SNpc), mice lacking *GRN* (*Grn* ^−/−^) showed an exaggerated, prolonged inflammatory response in activated microglia and that this mechanism likely contributed to enhanced neuron death following injury. Consistent with this, conditional mutants lacking *GRN* in microglia exhibited MPTP-induced phenotypes similar to *Grn* ^−/−^ mice. Selective depletion of *GRN* from microglia in mixed cortical cultures resulted in increased death of wild-type neurons in the absence of injury. Furthermore, *Grn* ^−/−^ microglia treated with LPS/IFN-*γ* exhibited an amplified inflammatory response and conditioned media from these microglia promoted death of cultured neurons. These results indicated that *GRN* deficiency leads to dysregulated microglial activation and thereby contributed to increased neuron loss with injury, revealing a role for *GRN* in the attenuation of neuroinflammation and suggesting that this mechanism contributed to neurodegeneration in *GRN-*deficient FTLD. Further, *GRN* may attenuate inflammation and neuron death in other forms of neurodegeneration or CNS injury [126]. 

 Interestingly, progranulin also seems to play a role in the activation of microglia in AD. *GRN* polymorphisms have been associated with AD [127, 128], and progranulin has been found to colocalize with A*β* plaques in brains of AD patients [129] and in some lines of transgenic mice models of AD [130]. Recently, Pickford et al. studied the effect of progranulin on neurons and microglia [131]. Using microarray and cytokine arrays, they found out that progranulin increased the secretion of leptin and Th2 cytokines, such as IL-4, IL-10, and IL-5, which have been associated with neuroprotection. Moreover, progranulin reduced the secretion of TRAIL, a member of the TNF superfamily, which is a regulator of apoptosis and is upregulated in the AD patients' brains [132]. Reduction of TRAIL secretion may be a neuroprotective mechanism of progranulin. They observed that progranulin promoted chemotaxis and endocytosis of extracellular peptides, including A*β*, by microglia. In addition, progranulin induced differentiation of microglia into the M2 anti-inflammatory phenotype. So, the activation of progranulin pathway could be a new strategy to promote microglial clearance of A*β* without the activation of cytotoxic cytokines [131]. 

 Bellucci et al. [4] focused instead their attention on a mutation of tau protein. In particular, they studied the effect of P301S mutated human tau protein in a patient with FTLDP-17T, a hereditary neurodegenerative disorder characterized by a spectrum of clinical phenotypes ranging from an FTLD-predominant to a parkinsonism-predominant type, linked to chromosome 17 with tau mutations [133]. In the cortex and hippocampus of the P301S patient, activated microglia, expressing CD68, and infiltrating macrophages were detected. These cells were particularly concentrated in the surroundings of phosphorylated (phospho)-tau-positive neurons. Moreover, the presence of CD68 positive cells around blood vessels indicated that the reactive microgliosis was probably accompanied by a remarkable macrophage infiltration. Activated microglia evolved also in a macrophagic state, a condition that happens during chronic neuroinflammation, and phospho-tau-positive neurons released activating signals, such as IL-1*β* and COX2. IL-1*β* acts as a chemotactic factor, and COX2 induces the production of prostaglandins, which may activate several intracellular kinases able to phosphorylate tau on specific sites [134]. Based on these results, the authors concluded that microglial activation and the production of proinflammatory mediators by phospho-tau-positive neurons may differentially contribute to neuronal death and disease progression in neurodegenerative tauopathies [4]. 

## 5. Conclusions

 In the last few years, genetic and molecular aspects at the basis of AD and FTLD have been better clarified, including the role of genes, inflammatory factors, and brain immune cells, microglia. Regarding autosomal dominant forms of AD and FTLD, many new genes have been identified, and new diagnostic criteria have been proposed. Concerning events at the basis of the diseases, it has become clearer and clearer that microglia play a crucial role, displaying both inflammatory and neuroprotective properties. 

Activated microglia could help in the recovery process or potentially aggravate CNS damage. In addition, a link has found between products of mutated genes, microglia, and inflammatory factors. A better understanding of such mechanisms, responsible for the development and progression of neurodegenerative disorders, will be a challenge for the future, with the aim of identifying specific targets for a tailored therapy. 
